# Positional Hemodynamic Instability in a Patient With Pectus Excavatum Undergoing Lumbar Discectomy

**DOI:** 10.7759/cureus.109406

**Published:** 2026-05-21

**Authors:** Izabella J Brykczynska, Francesco Carbone, Immanuel Höwelhaus, Augusto Leone, Uwe Spetzger

**Affiliations:** 1 Medicine and Surgery, Lazarski University, Warsaw, POL; 2 Neurosurgery, Riuniti Hospital, Foggia, ITA; 3 Neurosurgery, Karlsruhe Municipal Hospital, Kalrsruhe, DEU; 4 Anesthesiology, Karlsruhe Municipal Hospital, Karlsruhe, DEU; 5 Neurosurgery, Karlsruhe Municipal Hospital, Karlsruhe, DEU; 6 Neurosurgery, Institute for Anthropomatics and Robotics, Karlsruhe Institute for Technology (KIT), Karlsruhe Municipal Hospital, Karlsruhe, DEU

**Keywords:** cardiac abnormalities, complications, knee-chest position, patient care, pectus excavatum, spinal surgery

## Abstract

Pectus excavatum (PE) is the most common congenital chest wall deformity, accounting for 90% of cases, and may predispose patients to hemodynamic compromise due to cardiac compression. Such events during spinal surgery, however, are rarely reported. In the widely used genupectoral prone position for spine surgeries, the patient lies prone with the torso supported and the legs flexed, thereby reducing abdominal compression, improving venous drainage, and reducing bleeding. However, the support provided by the thorax in this position may cause significant mediastinal compression in the presence of pectus excavatum, potentially resulting in hemodynamic instability. In this case, a 65-year-old man experienced hemodynamic collapse more than ten minutes after positioning, following further optimization of thoracic support for lumbar discectomy. The procedure was halted, and the patient was repositioned supine, which promptly resolved the instability. Postoperative evaluation revealed a Type 2 myocardial infarction (NSTEMI) without significant coronary artery stenosis on delayed angiography, consistent with demand ischemia secondary to mechanically induced reduction in cardiac output. Given the risk of spinal epidural hematoma, the decision to delay anticoagulation is the most instructive point for spine surgeons and should remain central to this discussion. Although the clinical course was ultimately benign, neurosurgeons and anesthesiologists should remain aware of the potential complications associated with the knee-chest position in patients with significant pectus excavatum. A thorough physical examination is therefore essential before positioning a patient, even for standard procedures.

## Introduction

Pectus excavatum (PE) is the most common congenital deformity of the anterior thoracic wall, occurring in approximately one in every 400 to 1,000 live births [1]. The condition is more prevalent among individuals with connective tissue disorders such as Marfan syndrome and is frequently associated with scoliosis [2]. The CT-derived Haller Index remains the “gold standard” for assessing deformity severity preoperatively [2]. When scoliosis is present, the sternum is often displaced from the midline of the vertebral column. Hemodynamic compromise may arise from both the prone positioning required during posterior spinal surgery, which increases anterior cardiac compression, and from anterior shifting of the right-convex thoracic spine toward the left, which can exacerbate posterior cardiac compression [3]. In the knee-chest position, obstructive physiology "forces" the sternum closer to the spine under gravity and external support compared with the prone position. The weight of the chest is often supported by bolsters, which increases the sternum's posterior displacement towards the heart. Interestingly, while PE is typically managed in younger patients, treatment in older individuals is further complicated by increased chest wall rigidity and the potential coexistence of coronary artery disease, which can significantly obscure the clinical picture [4].

Despite growing recognition of the cardiopulmonary implications of PE, the published literature contains fewer than five case reports over more than two decades describing intraoperative hemodynamic compromise attributable to positional mechanical cardiac compression in patients with PE [3,5-8]. Critically, all previously reported cases share three defining features that the present case does not: (1) they occurred exclusively in paediatric or adolescent patients (age range: 13 months to 17 years); (2) PE was in each instance associated with scoliosis and, in the majority of cases, with a connective tissue disorder (Marfan syndrome) or neurofibromatosis; and (3) the precipitating position was standard prone positioning during posterior spinal fusion surgery.

We report a rare case of a 65-year-old male patient with PE who developed acute cardiovascular decompensation after positioning in the genupectoral (knee-chest) position for minimally invasive spinal surgery. Repositioning to the supine position resulted in rapid hemodynamic stabilization. Subsequent evaluation revealed a Type 2 myocardial infarction (Type 2 MI), as defined by the fourth universal definition of myocardial infarction [9]. The myocardial injury arose from an acute oxygen supply-demand imbalance unrelated to coronary atherothrombosis. The mechanism in our patient was a sudden, mechanically induced reduction in cardiac output caused by right atrial compression from the depressed sternum, which lowered coronary perfusion pressure below the ischemic threshold. This interpretation is supported by a dynamic troponin rise above the 99th percentile, new ECG changes, CT-confirmed direct sternum-right atrium contact, and the absence of significant coronary artery stenosis on delayed angiography.

The present case is, to our knowledge, the first to describe this mechanism in an adult patient with isolated PE and no syndromic comorbidity, in the genupectoral (knee-chest) prone position during minimally invasive lumbar surgery

## Case presentation

Clinical presentation

We present the case of a 65-year-old man who was admitted to the emergency department with a five-day history of lumboischialgia and high-grade paresis of the left foot. Magnetic resonance imaging (MRI) demonstrated a left lumbosacral sequestrated disc herniation (Figures 1, 2). His medical history included a pericardial effusion following prior pulmonary vein ablation for atrial fibrillation, hyperlipidemia, and carotid atherosclerosis. The atrial fibrillation had been successfully terminated by ablation, and there was no evidence of ongoing anticoagulation therapy according to medical records and patient history. Physical examination also revealed a notable pectus excavatum deformity, which had not previously caused symptomatic cardiopulmonary limitation. On neurological examination, sensory function was preserved, but gait was unstable on the left side. Deep tendon reflexes were slightly diminished bilaterally. Strength testing revealed severe paresis [Medical Research Council (MRC) 1/5] of the left tibialis anterior and complete paralysis (MRC 0/5) of the extensor hallucis longus. No deficits were observed on the right side. Routine blood tests showed no new abnormalities, aside from a known mild thrombocytopenia (146/nL). Given the acute presentation with high-grade distal paresis, emergency surgery was indicated.

**Figure 1:**
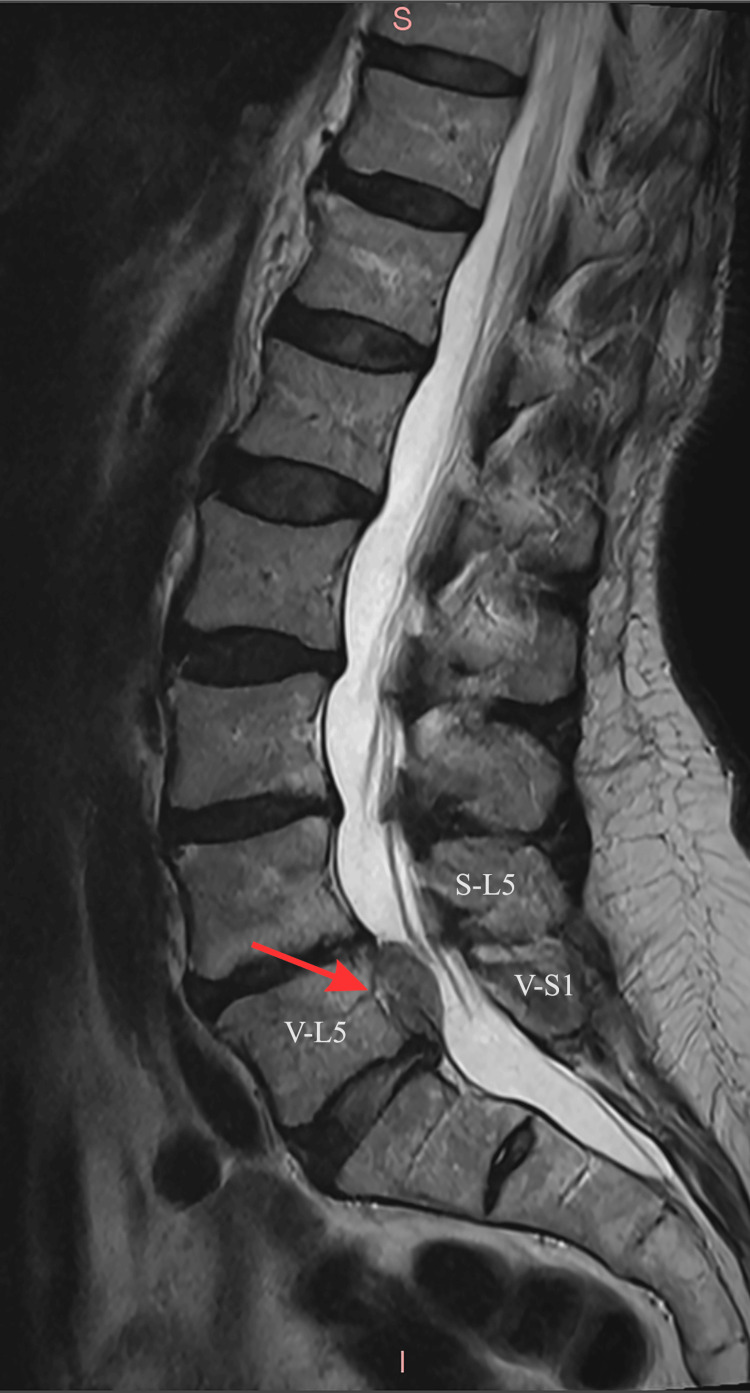
Sagittal T2-weighted MRI of the lumbar spine, demonstrating a large cranially-migrated left-sided disc herniation at the L5-S1 level. S-L5: Sacrum to 5th Lumbar Vertebra; V-L5: 5th Lumbar Vertebra; V-S1: 1st Sacral Vertebra

**Figure 2:**
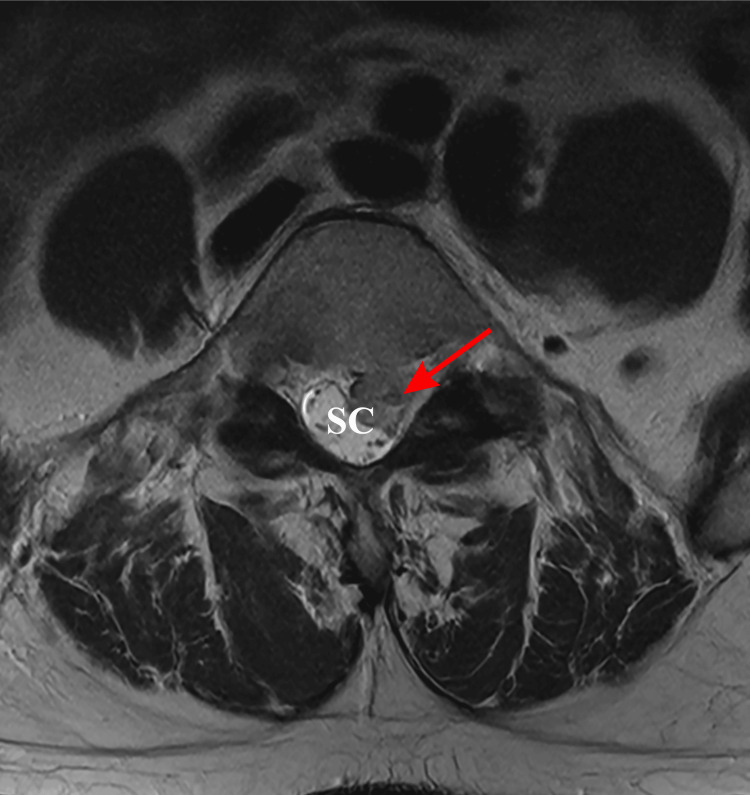
: Axial T2-weighted MRI of the lumbar spine, demonstrating a large cranially-migrated left-sided disc herniation at the L5-S1 level. SC: Spinal Canal

Intraoperative findings

During standard microsurgical positioning in the knee-chest prone position to widen the interlaminar window, the patient initially remained hemodynamically stable after turning prone but exhibited unacceptable cervical hyperextension. Additional thoracic padding was therefore placed. Approximately 10-12 minutes after positioning, the patient developed sudden cardiovascular collapse with profound hypotension, and a sudden drop of endtidal CO² as shown in Figure 3. The patient was immediately repositioned supine, and bolus catecholamines were administered for 30 minutes, followed by a short continuous infusion of noradrenaline. Focused transthoracic ultrasound demonstrated transiently reduced ventricular systolic function, which normalized within approximately 30 seconds. Continuous intra-arterial blood pressure monitoring was subsequently established. After exclusion of lower-limb deep venous thrombosis by duplex sonography and confirmation of normalized cardiac function, an interdisciplinary decision was made to proceed with surgery. Venous air embolism was excluded due to the rapid normalization of hemodynamic parameters with repositioning, and there was no evidence of deep vein thrombosis. To improve hemodynamic safety, the operation was continued in a flat prone position, thereby eliminating cervical hyperextension and avoiding further thoracic over-padding. The procedure was completed successfully, and the patient was transferred to the intensive care unit (ICU) for close postoperative monitoring.

**Figure 3:**
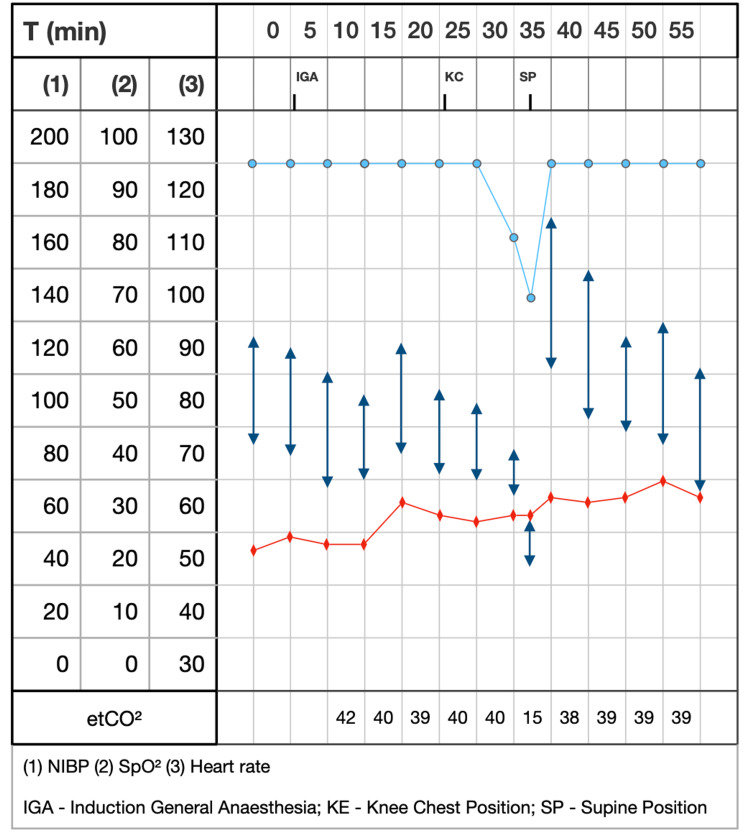
Time-dependent evolution of the patient’s hemodynamic parameters. NIBP: non-invasive blood pressure; SpO2: Peripheral oxygen saturation.

Postoperative care

Postoperatively, laboratory testing revealed a marked rise in high-sensitivity troponin I (hsTnI) from 95 to 776 ng/L, compatible with acute infarction. Electrocardiography showed QTc prolongation (499 ms), reflecting the electrophysiological abnormalities often induced by acute myocardial ischemia and indicating increased risk of arrhythmias. Computed tomography excluded pulmonary embolism but demonstrated direct contact between the depressed sternum and the right atrium even in the supine position, supporting the hypothesis that additional thoracic pressure in the knee-chest position caused critical right atrial compression. This provided a plausible structural explanation for the abrupt, position-dependent intraoperative hemodynamic collapse. Based on these findings, a diagnosis of NSTEMI was made according to current guidelines [4,5].

Given the recent spinal surgery and mild clinical presentation, coronary angiography was initially deferred to avoid the postoperative hemorrhagic risk associated with anticoagulation. Although early angiography may reduce recurrent ischemic events, immediate high-dose heparin was considered to carry an unacceptably high risk of spinal rebleeding and permanent neurological injury. As the patient remained hemodynamically stable in the ICU, a conservative strategy was adopted.

After extubation, cardiopulmonary status stabilized (heart rate 69 bpm), with normal creatine kinase (CK) and CK-myocardial band (MB) levels. On postoperative day one, following cardiology consultation, aspirin and prophylactic certoparin were initiated. Neurological examination showed partial improvement (left tibialis anterior MRC 3/5; extensor hallucis longus MRC 1/5). Transthoracic echocardiography was unremarkable.

By postoperative day three, ECG findings had normalized, and hsTnI levels declined. The patient was transferred to the neurosurgical ward, where further neurological recovery was observed (tibialis anterior 4/5; extensor hallucis longus 3/5) with stable cardiopulmonary status. The patient was discharged on postoperative day 4. One month later, coronary angiography demonstrated only mild coronary sclerosis without significant stenosis, supporting the proposed pathophysiological mechanism for the NSTEMI, resulting from mechanical obstruction. Aspirin was continued, and the patient remained neurologically and cardiopulmonary stable at follow-up.

## Discussion

This case illustrates a rare but potentially devastating complication of spinal surgery due to cardiovascular collapse during genupectoral positioning in a patient with PE and multiple cardiovascular risk factors. The abrupt onset with severe hypotension, resolving immediately upon supine repositioning, strongly suggested a structural mechanism - most likely compression of the right ventricle with impaired venous return. As a low-pressure structure, the right ventricle is extremely vulnerable to external mechanical pressure, resulting in an immediate decrease in preload and cardiac output. The delayed onset of hemodynamic collapse after more than ten minutes in position, together with immediate recovery after supine repositioning, argues strongly against simple venous pooling and supports a mechanical thoracic compression mechanism. This interpretation aligns with previous reports demonstrating that prone or genupectoral positioning can exacerbate pre-existing cardiopulmonary compromise in patients with severe chest wall deformities [10].

Pathophysiology of positional hemodynamic collapse in pectus excavatum

PE is the most common congenital chest wall deformity, accounting for up to 90% of thoracic malformations. Clinically significant cardiopulmonary complications are relatively uncommon, making reports of intraoperative cardiovascular events particularly noteworthy. PE usually develops in childhood or adolescence and may progress with age [7,8]. Although it does not typically reduce life expectancy, it can cause substantial functional and psychosocial burden [4]. While often asymptomatic, severe PE may present with exercise intolerance, exertional chest pain, restrictive lung function, cardiac displacement, and mitral valve abnormalities [7,11]. Patients may also experience shortness of breath, recurrent respiratory infections, or the so-called “pectus posture” characterized by thoracic kyphosis, forward shoulders, and a protuberant abdomen. Cardiac manifestations include murmurs, mitral valve prolapse, and right ventricular compression. Pulmonary function tests may reveal reduced forced vital capacity (FVC), forced expiratory volume (FEV)1%, and forced expiratory flow (FEF)25-75%. Moreover, PE is associated with connective tissue disorders such as Marfan syndrome [2].

The primary hemodynamic collapse mechanism in our patient was mechanical right atrial compression. Four convergent lines of evidence support this as the dominant pathophysiology. First, postoperative CT demonstrated direct contact between the posteriorly displaced sternum and the anterior wall of the right atrium at rest in the supine position (Figure 4), establishing a pre-existing structural vulnerability to any additional compressive load. Second, the delay of 10-12 minutes between prone positioning and hemodynamic collapse argues against acute venous pooling or anesthetic-related vasodilatation and is instead consistent with a progressive accumulation of compressive force, most plausibly exerted by the supplemental thoracic padding placed to correct cervical hyperextension. Third, immediate and complete hemodynamic normalization upon supine repositioning confirms that the mechanism was positional and reversible. Fourth, the absence of compensatory tachycardia during the hypotensive event is inconsistent with hypovolemia and supports a fixed mechanical obstruction to cardiac output, as impaired ventricular filling from right atrial compression prevents the cardiac output response that would normally trigger baroreceptor-mediated tachycardia.

**Figure 4:**
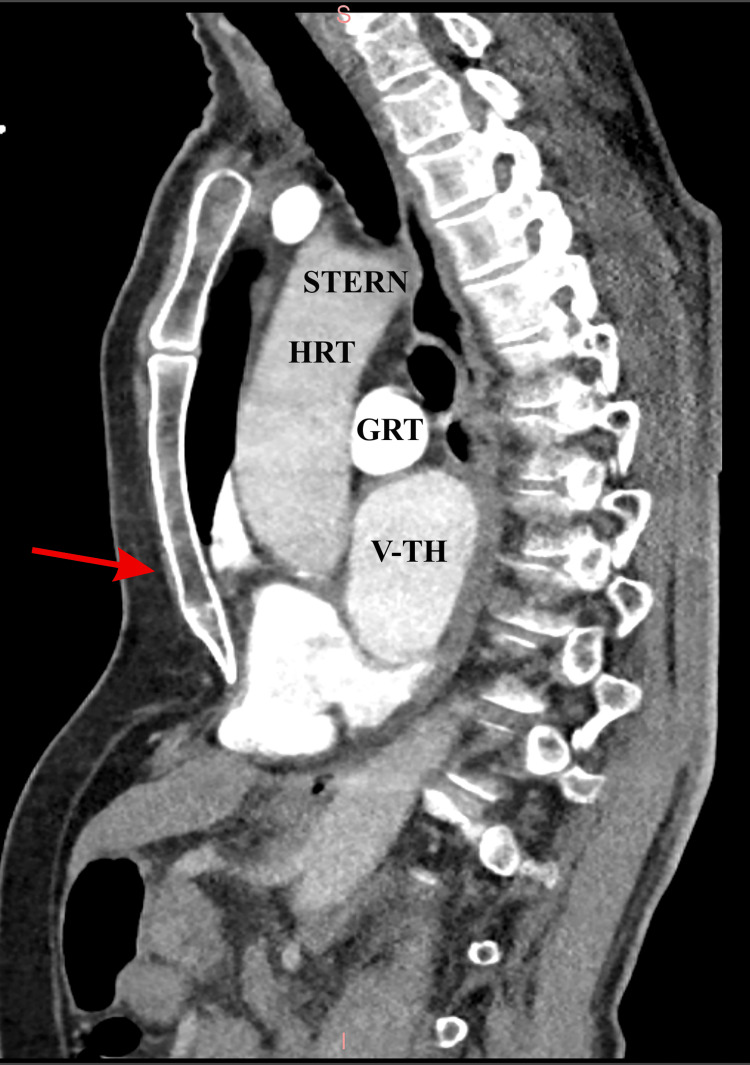
Sagittal contrast-enhanced CT of the thorax demonstrating a posterior displacement of the sternum consistent with PE, resulting in compression and displacement of the heart and great vessels within the mediastinum. STERN: Sternum; HRT: Heart; GRT: Great Vessels; V-TH: Thoracic Vertebra

Although a concomitant vagally mediated component cannot be excluded, particularly given the non-physiological cervical hyperextension present at the time of collapse. This is considered a secondary and contributory mechanism rather than a primary driver, and the clinical resolution pattern is most consistent with a structural, mechanically reversible cause.

Comparison with published cases

Five published case reports have been published so far, describing intraoperative or perioperative hemodynamic compromise from positional mechanical cardiac compression in patients with PE undergoing spinal surgery (Table 1). While each case carries unique clinical characteristics, all share the same fundamental pathophysiological pattern: sternal depression creates a vulnerable thoracic geometry in which prone positioning (particularly with transverse thoracic support) generates critical compression of the right-sided cardiac chambers, acutely reducing venous return and cardiac output. Alexianu et al. and Bafus et al. were the first to confirm this mechanism directly by intraoperative transesophageal echocardiography (TEE), demonstrating right ventricular compression by the sternum immediately upon prone positioning, with TEE resolution when longitudinal bolsters replaced transverse chest pads [5,6]. Adachi et al. and Uvodich et al. extended this understanding by documenting a dual compression mechanism in which posterior spinal correction further narrows the mediastinum, worsening the Haller index and making hemodynamic instability more refractory and difficult to reverse after surgery [3,7]. Xu et al. [8] contributed an important monitoring insight: a progressive fall in end-tidal CO2 (PETCO2) may precede overt arterial hypotension as the earliest sign of obstructive shock from reduced pulmonary blood flow from right heart compression, underscoring the value of multi-parameter intraoperative vigilance. For instance, the abrupt precipitation of end-tidal carbon dioxide (EtCO2), as seen in Figure 3, supports the pathophysiological theory of mechanical obstruction of the inferior vena cava or right atrium in the present case.

**Table 1:** Summarizing table of previously reported cases of cardiovascular collapse during spinal surgery in the prone position in patients with PE. PE: pectus excavatum; TEE: trans-esophageal echocardiography; PETCO2: progressive fall in end-tidal CO2; RA: right atrium; RV: right ventricle.

	Alexianu et al. [4]	Bafus et al. [5]	Adachi et al. [3]	Uvodich et al. [6]	Xu et al. [7]	Present case
Age/sex	34 months, male	15 years, male	13 years, male	16 years, male	17 years, female	65 years, male
Background	Neurofibromatosis, scoliosis, PE	Scoliosis, PE, marfanoid habitus	Marfan syndrome, scoliosis, PE	Marfan syndrome, scoliosis, severe PE	Idiopathic scoliosis, PE	PE without scoliosis/Marfan
Surgery	Posterior spinal fusion	Posterior spinal fusion	Posterior spinal fusion	Posterior spinal fusion	Posterior spinal fusion	Lumbar discectomy
Position during surgery	Prone on transverse bolsters	Prone on the Jackson table	Prone, worsened during thoracic correction	Prone	Prone with a transverse chest pad	Knee-chest prone with added thoracic padding
The main early sign	Severe hypotension	Severe hypotension	Severe hypotension and tachycardia	Severe hypotension and tachycardia	Progressive fall in PETCO2, then hypotension	Severe hypotension without tachycardia
Mechanism	RV compression by sternum on TEE	RA-RV inflow obstruction on TEE	RA-RV compression	RA-RV compression	RA-RV compression	RA compression from PE, likely worsened by thoracic support in the knee-chest position
Response to supine repositioning	Immediate recovery	Immediate recovery	Immediate recovery	Incomplete immediate recovery; prolonged postoperative shock	Immediate recovery	Immediate recovery

Perioperative management and risk stratification

Our patient presented with acute lumboischialgia and rapidly progressive left foot paresis. During emergency lumbar microsurgery in the genupectoral position, he developed severe hypotension requiring urgent supine repositioning. Although prone positioning is routinely used in spine surgery, it can increase intrathoracic compression, reduce cardiac output, and precipitate hemodynamic compromise in patients with anterior chest wall deformities [3]. Literature documenting intraoperative cardiovascular collapse due to PE remains scarce, with available reports summarized in Table 1. In our patient, the critical precipitant was not prone positioning per se, but the addition of supplemental thoracic padding to correct cervical hyperextension. This detail has important practical implications: preoperative and postoperative echocardiography were unremarkable. CT evidence of sternum-right atrium contact was only available postoperatively.

Based on the reviewed literature and the present case, we propose that patients with PE scheduled for elective spinal procedures should be stratified as high perioperative risk if there is evidence of associated connective tissue disorder (e.g., Marfan syndrome) or planned spinal deformity correction with anticipated postoperative increase in mediastinal compression. However, preoperative recommendations (e.g., a CT scan of the thorax) or echocardiography are not recommended, as they would expose the patient to radiation risk or yield limited findings. Nevertheless, a thorough physical examination and explicit recognition of PE should trigger heightened intraoperative vigilance and avoidance of supplemental thoracic padding. In patients with known PE, physical examination remains the most important factor in choosing the right surgical table (e.g., use the Jackson table or the Wilson frame instead of the Andrews frame to avoid compressing the sternum) [12].

Limitations

This report carries several inherent limitations that must be acknowledged. First, as a single case report, the findings are anecdotal by nature and cannot be generalized to the broader population of patients with PE undergoing spinal surgery. Second, continuous intra-arterial blood pressure monitoring was initiated only after hemodynamic collapse, as its placement was not requested in this brief, minimally invasive procedure; the hemodynamic values reported for the peri-collapse period are therefore approximate estimates derived from non-invasive monitoring records. Third, intraoperative echocardiography was not available at the time of the acute event, precluding real-time visualization of right atrial compression and formal quantification of its hemodynamic consequences. Finally, coronary angiography was appropriately deferred due to the unacceptably high hemorrhagic risk of immediate anticoagulation in the early postoperative period following spinal surgery; while the delayed finding of only mild coronary sclerosis without significant stenosis strongly supports a Type 2 MI mechanism, a transient acute coronary syndrome as a concurrent contributing trigger cannot be entirely excluded.

## Conclusions

This case demonstrates that PR can precipitate acute, life-threatening cardiovascular collapse during spinal surgery even in adults without scoliosis or connective tissue disorders, and even in standard positions such as the genupectoral posture. The position-dependent onset, absence of compensatory tachycardia, immediate resolution upon supine repositioning, and CT-confirmed sternum-right atrium contact collectively identify mechanical right atrial compression as the primary mechanism. The resulting Type 2 MI represents a previously unreported complication in this context. The objective anatomical assessment revealed a Haler Index of 3.3, which exceeds the clinically established threshold of 3.25 for significant pectus excavatum. In contrast to the simple prone position on a Jackson table, the knee-chest position poses the greatest risk, as it concentrates all the weight of the chest on the support points. A drop in EtCO2 served as a warning sign observed prior to complete cardiac arrest.

For neurosurgeons and anesthesiologists, this underscores the importance of thorough preoperative assessment in patients with chest wall deformities or cardiovascular comorbidities. Intraoperatively, invasive monitoring and preparedness for urgent repositioning are essential safeguards. When feasible, alternative positioning strategies or staged correction of severe chest wall deformities should be considered to mitigate perioperative risk.
